# Interactomes, manufacturomes and relational biology: analogies between systems biology and manufacturing systems

**DOI:** 10.1186/1742-4682-8-19

**Published:** 2011-06-20

**Authors:** Edward A Rietman, John Z Colt, Jack A Tuszynski

**Affiliations:** 1 Center for Cancer Systems Biology, Dana Farber Cancer Institute, Boston MA, 02115, USA; 2 Department of Genetics, Harvard Medical School, Boston, MA, 02115, USA; 3 IBM Corporation, Essex Junction, VT, 05452, USA; 4 Department of Experimental Oncology, Cross Cancer Institute, 11560 University Av. Edmonton, AB, T6G 1Z2, Canada; 5 Department of Physics, University of Alberta, Edmonton, AB, T6G 2G7, Canada; 6 Center of Cancer Systems Biology, St. Elizabeth's Medical Center, Tufts University School of Medicine, Boston, MA, 02135, USA

## Abstract

**Background:**

We review and extend the work of Rosen and Casti who discuss category theory with regards to systems biology and manufacturing systems, respectively.

**Results:**

We describe anticipatory systems, or long-range feed-forward chemical reaction chains, and compare them to open-loop manufacturing processes. We then close the loop by discussing metabolism-repair systems and describe the rationality of the self-referential equation *f *= *f *(*f*). This relationship is derived from some boundary conditions that, in molecular systems biology, can be stated as the cardinality of the following molecular sets must be about equal: metabolome, genome, proteome. We show that this conjecture is not likely correct so the problem of self-referential mappings for describing the boundary between living and nonliving systems remains an open question. We calculate a lower and upper bound for the number of edges in the molecular interaction network (the interactome) for two cellular organisms and for two manufacturomes for CMOS integrated circuit manufacturing.

**Conclusions:**

We show that the relevant mapping relations may not be Abelian, and that these problems cannot yet be resolved because the interactomes and manufacturomes are incomplete.

## Background

Systems biology is a domain that generally encompasses both large-scale, organismal systems [1], and smaller-scale, cellular systems [2]. The majority of contemporary systems biology falls under the cellular-scale studies with the large goals of understanding genome to phenome mapping. This cellular-scale, or molecular systems biology, may also contribute to synthetic biology by becoming the theoretical underpinning of that, largely, engineering discipline; and it may also contribute to a perennial question of physics - the difference between living and non-living matter. It is this latter question that concerns us in this paper.

There is significant other research focusing on defining the difference between living and nonliving matter. These including: category theory [3,4], genetic networks [5], complexity theory and self-organization [4-7], autopoiesis [8], Turing machines and information theory [9], and many others that are not reviewed here. It would take a full-length book to review the many subjects that already come into play in discussing the boundaries between living and nonliving. Here we concern our self only with factory system analogies and cellular molecular networks, as we explore the boundaries that define life.

Several disparate mathematical and analytical techniques have been brought to bear on defining and analyzing molecular network systems [10,11]. For example, Alon [12] focuses on understanding the logic of small-scale biomolecular networks; Kaneko [2] studies systems biology from a dynamical systems point including molecular, cellular development and phenotypic differentiation and fluctuations; Huang et al. [13] considers the gene networks from a dynamics perspective, in particular as dynamic landscapes settling to attractor states and limit cycles; and Palsson [14], focus on metabolic and biochemical networks using very large systems of differential and difference equations. Fisher and Henzinger [15] have reviewed other mathematical methods, such as Petri nets, Pi calculus and membrane computing.

The Petri net approach to systems biology is reasonable and draws on analogies from manufacturing systems [15-17]. Armbruster et al. [18] outline and describe the similarities between networks of interacting machines in factory production systems and cell biology, and Iglesias and Ingalls [19] describe analogies between control theory and systems biology. Casti [20,21] makes mathematical analogies between factory systems, control theory and connects it to cellular biology via a set of mathematical tools known as category theory. The primary, and still the main work, on category theory to biology is Rosen [3,22,23]. He defines it as *relational biology*.

Relational biology, as defined by Rosen [3], is a mathematical exploration of the principles, of the boundary between living and non-living phenomena. His approach was based on category theory. Our exploration of this area of relational biology will draw on analogies between factory systems and biological systems. Our primary references for that section of our review will be Casti [20,21].

## Main text

### Anticipatory Systems

**Figure 1:**
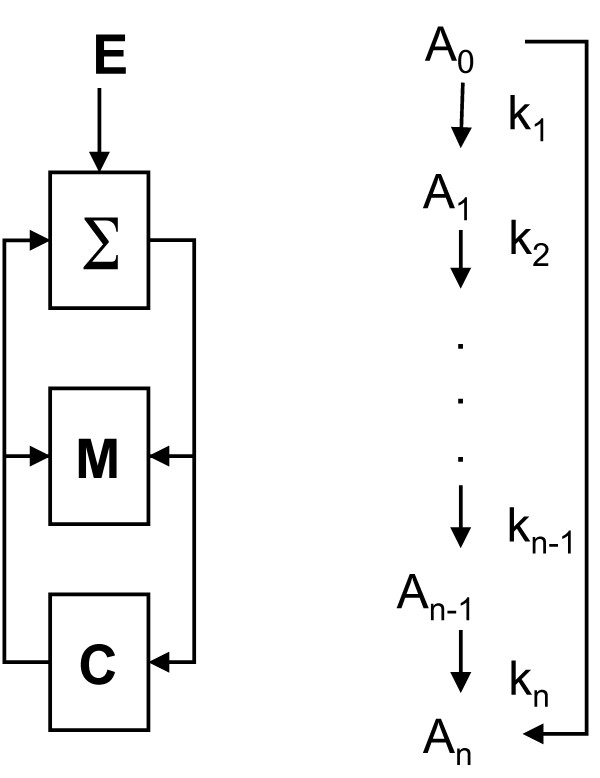
**Flow diagram of an anticipatory system (left), and a simple chemical reaction network diagram (right)**.

At a fundamental level cells, like factory production systems contain anticipatory systems, and much of the mathematics associated with factories can be exploited for systems biology. We start by analyzing the feed-forward system known as the coherent feed-forward loop described by Alon [12] and Mangan et al. [24]. It is a very common network motif in molecular system networks. An abstract example of the arabinose system of *Escherichia coli *is shown in Figure 1. Another example is the MAP kinase cascade. These are known as anticipatory systems and contain within themselves models of the system and the system controller. The phrase anticipatory system, by itself, seems to ignore causality. But in fact the causality is preserved by the fact that the model uses information from prior system states to predict future states. These anticipatory systems are said to be able to anticipate the future, but as we will see, these systems contain implicit system models of process controllers that enable them to seemingly anticipate the future. Because there is no explicit model, the actual process being controlled can drift in performance due to subsystem changes.

Figure 1 shows a flow diagram of an anticipatory system. The only assumptions in this model are that each chemical species is "processed" by a unique enzyme to produce another chemical species. The environment, *E*, sends signals to the system, *∑*. The model, *M*, reads the state of the system. The controller, *C*, sends signals to the system and the model. Causality is preserved by the fact that the past influences the prediction.

As an example of an anticipatory system consider the chemical reaction network shown in Figure 1. A chemical substrate *A_i _*is in the reaction sequence at *i*. The rate of the chemical reaction, or conversion of *A_i _*to *A*_*i+*1_, is given by *k*_*i+*1_, and *i *= 1,2, ...,*n *are the individual molecular substrates. The reaction from *A*_0 _→ *A_n _*is known as a forward activation step. Concentration of *A*_0 _activates the production of *A_n_*. In other words, concentration of *A*_0 _at *t predicts *concentration of *A_n _*at *t *+ *τ*. Essentially then, *k_n _*= *k_n _*(*A*_0_) and we leave all other *k_i _*constant.

The reaction rates for the system can now be written as:

The forward activation step stabilizes the level of substrate *A*_*n*-1 _in the face of environmental fluctuations to the initial substance, *A*_0_. This stabilization is achieved through the relation:

This shows that stabilization is independent of *A*_0_, and we can write the rate equation for this as *k*_*n*-1 _*A*_*n*-2 _= *k_n _*(*A*_0_) *A*_*n*-1_. This relationship can be achieved by the linear system:

In this system, *K*_1 _and *K*_2 _are functions of the rate constants, *k_i_*, *i = *1,...,*n - *1. This clearly shows that *A*_0 _determines the values of *A*_*n*-1 _and *A*_*n*-2 _at future times. The control condition for *k_n _*(*A*_0_) must show that the rate for any step at any given time point be determined by the value of *A*_0 _at a prior time:

Given the fact that there is some production time associated with any given protein (i.e. kinetics), this model provides insight into a possible system stabilization mechanism, in the face of either environmental fluctuations and/or gene expression variability. This could explain the reason that "higher" organisms have a longer signaling cascade than bacteria. In this model homeostasis is preserved by the anticipation or prediction of *A*_*n*-1_. This is known as open-loop control, in engineering, because the system controller feeds into the process to be controlled without any signals feeding back from the process to the controller. The hazard in this type of control is it can result in global system failure.

To describe the weakness of open-loop control, or feed-forward control, assume our system, ∑ (e.g. factory or cell) is composed of *N *subsystems. The following input/output relation can give the behavior of any one subsystem *S_i _*:

The input is represented as *u_i _*and spans a real *m*-space. The output is represented as *y_i _*and spans a real *p*-space. The output from the subsystem is, of course a future time, represented as *t+h*, and the input occurs at time *t*. The subsystem can receive inputs either from other subsystems or from external sources.

The subsystem *S_i _*operates according to the function *φ_i _*(*u*_*i *_(*t*), *y*_*i *_(*t *+ *h*)), and is behaving well when the input and outputs are within the specified space (*u*_*i*_, *y_i_*) ∈ *R^m ^× R^p^*.

Analogously, the overall system ∑ has its own inputs, *ν *∈ *R^n ^*and output(s) *ω *∈ *R^q ^*relations that exist in some space Ω ⊂ *R^n ^*× *R^q^*. In order to evaluate the health of the system (factory or biological cell) there are four logical possibilities:

1. Each subsystem *S_i _*is operating optimally, therefore the global system ∑ is operating optimally.

2. The global system is operating optimally, therefore each subsystem is operating optimally.

3. Any subsystem failure gives rise to global system failure.

4. The health of a subsystem is not related to the health of the global system.

**Figure 2:**
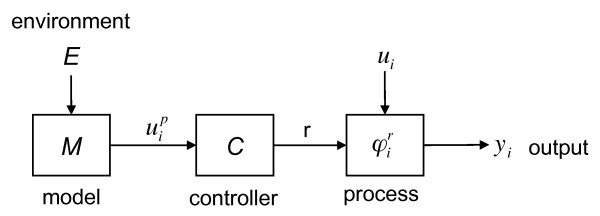
**Block diagram details of subsystem, SI**.

The fourth possibility we will reject as being unreasonable for real-world systems. The third possibility is valid only if there are no redundancies in the global system; again not realistic for either cells or real world factories. The first possibility is the opposite of possibility number two, which we will describe in detail and is referenced in Figure 2 for subsystem *S_i_*.

The input to the model, *E*, is from the environment. The model output, the predicted input for the process, is sent to the controller. The output from the controller, *r*, is the control vector and is sent to the process, as are other inputs from other subsystems. It is important to realize that the process,  governs the subsystem, *S_i _*which processes its input, *u_i _*(*t *+ *h*) at a later time *t *+ *h*.

Correct behavior of the global system ∑, indicates that the inputs and outputs lie within an acceptable region of Ω. For proper functioning of the global system,  must be adapting properly in the feed-forward loop. This proper functioning depends on the fidelity of model *M*. If the model is not updated from internal process signals then at some point the model will no longer be correct. Real world processes will have subsystems that degrade. This will result mean that the controller, and thus the model, are no longer commensurate with reality. In general there will be a time, *T*, at which this is no longer the case. *M *will effectively drift away from ideal behavior because there are no updates to the model. At this point the process *φ_i _*is said to be incompetent. For a linear anticipatory system this will lead to ∑ system failure.

Biological cells are excellent examples of systems that contain internal models of themselves. Biology adapts to this lack of model fidelity in feed-forward networks by a repair function. Basically, a cell has two related process, metabolism and repair. Let *A *represent a set of environmental inputs to the cell and *B *represent a set of output products. Then the set of physically realizable metabolisms is given by *H*(*A*, *B*). We can write the metabolic map as *f *: *A *→ *B*. We assume for the sake of argument that this map is bijective, so elements of the two sets map to each other *a *⟼ *b*.

Biology solves the model fidelity problem either by subsystem repair, or in some cases apoptosis - discard the system and start over. The repair operation *R*, is designed to restore metabolism *f*, when a particular environmental variable, *a *is a fluctuating time-series. This may involves synthesis of several enzymes and/or promoters to induce gene expression. Since we are assuming bijection and *a *⟼ *b*, then the subsystem output *y *must also be a fluctuating time-series. When the overall system is operating correctly the metabolism function, *f *operates on the time-series of all inputs *A *to produce the relevant time-series output set *B*. If the input does not fluctuate from the evolved basal metabolism, the "design space," then the repair function essentially produces more of the same: *R*: *B *→ *H *(*A*, *B*). This says that the repair function uses output *Y *from prior steps to produce a new metabolic map *H*. The boundary conditions for the metabolism and repair system are: *R*(*f *(*a*)) = *R *(*b*) = *f*. The repair operation is thus to stabilize any fluctuations in inputs or metabolism. The repair system, *R *is an error correcting mechanism. But when it fails the biological solution to the problem is to reproduce a new cell and destroy the broken one.

If a critical subsystem *S_i _*within the global system ∑ fails, then the cell signals to begin replication. This affectively solves the open-loop control problem of model drift. The cell's genome receives information about the metabolic system, *f *and builds a copy of repair system, *R*. This reproduction mapping relation is given by: *β *: *H*(*A*, *B*) → *H*(*B*, *H*(*A*, *B*)). This is summarized as:

Through metabolism, environmental signals are converted into cellular outputs and subsystem outputs. These signal the translation apparatus to begin building a new metabolism system. These "self-referential" systems are known as metabolism-repair systems (M-R) systems and can be described with category theory.

Among others, real biological examples of the anticipatory systems include the flagella motor expression in *E. coli *[25] and part of the hepatocytes regulatory network [26].

### M-R Systems and Category Theory

**Figure 3:**
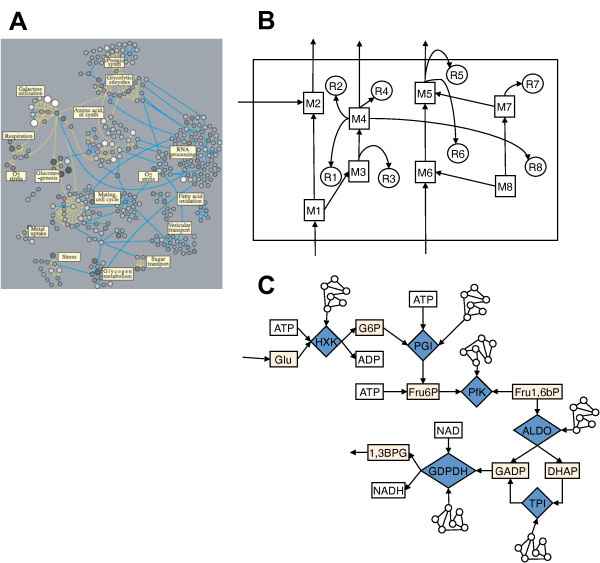
**Network and block diagrams**. Panel A: Diagram from Ideker et al [27] of a segment of genomic-proteomic-metabolic network. Panel B: A simplified block diagram of an M-R system. Panel C: Partial block diagram of glucose metabolism system.

Rosen [3] summarized decades of his research on anticipatory and M-R systems, in his book: *Life Itself, A Comprehensive Inquiry into the Nature, Origin and Fabrication of Life*. There, he used extensively a branch of mathematics known as category theory, a theory involving mappings of sets and functions. To describe an M-R system we consider a simple model consisting of metabolism and repair "components." Each *M_i _*and *R_i _*is a considered as a closed black box. Figure 3A shows a genomic-proteomic-metabolic network from Ideker et al. [27], and Figure 3B shows a simplified M-R system block diagram. As seen in the block diagram each M-block has associated with it an R-unit. If for example, subsystem M6 fails then a signal from M5 will activate the R6 unit to begin building a new M6 unit. This scheme will work only if M5 has already produced a threshold level of R6 components. Otherwise since M5 is linked to M6 the entire pathway of M6-M5 could fail. Now consider M2, if it fails M4 can produce a new R2 unit. Notice that M1 is also connected to M4 so there is a complete path from the input at M1 to the output at M4 via M3, and thus the synthesis of R1 the repair unit for M1. This dependency relation in these M-R system models is exactly the same as anticipatory systems described above. M5 is the weaklink in the system. It is not a repairable component. When it fails, apoptosis will be invoked.

The concept of non-repairable molecular components in cells of course is not new. Hillenmeyer et al. [28] preformed knockout experiments on yeast, and showed that many genes, causes little or not phenotypic effects in multiple chemical environments. Clearly, this indicates massive redundancy in the genomic, and thus the proteomic, networks. The network diagram in Figure 3A shows some of the potential redundancy. The nodes in this network are genes. The yellow connections between genes indicate that protein encoded by one of the genes binds to the second gene (protein → DNA). The blue lines indicate a direct protein-protein binding. As shown by Hillenmeyer et al. [28], the actual number of critical genes in the yeast network is only about 20%.

For M-R systems the equation *β*: *H*(*A*, *B*) → *H*(*B*, *H*(*A*, *B*)) should not represent reproduction, per se, but rather re-synthesis, and the diagram in Figure 3B should show some metabolic closure. To a first order, life is a complex self-replicating chemical network enclosed in a self-synthesized membrane that allows specific external molecular substrates to enter the network and other molecular species to exit the network. To describe this in more detail, consider Figure 3C. Here we see a segment of the glucose utilization pathway. The diamonds in the flowchart are enzymes or, in terms of manufacturing systems, they are the small machines that take inputs and produce outputs. For example HXK processes ATP and Glucose to produce G6P and ADP. Similarly, PGI accepts G6P and additional ATP to produce Fru6P. Other segments are similarly interpreted. These processing units in the network are said to be components of the metabolism network, while all the components in rectangular boxes are inputs and outputs to these machines.

Adapting some terminology from Letelier et al. [29,30], we will represent the entire set of processing machines, or enzymes, as the set {*M*}. While the entire set of inputs and outputs are represented as {*A*} and {*B*} respectively. We thus have the mapping relationship *M *: *A → B *representing *all possible *mappings from inputs to outputs.

Figure 3C also shows small network icons connecting to the *M*, diamonds. Real enzymes degrade or need to be replaced. In Rosen's terminology, the broken or failing *M *units are repaired. Each *M_i _*has associated with it a repair unit, *R_i_*, so there is an entire set of repair units, {*R*}. In biological systems the repair would simply be replacement. This replacement is how biological systems circumvent the open-loop control found in so many subsystems (or subnetworks). We represent the *R_i _*units as network icons to remind us that the actual repair or replacement comes about as a result of a network of subreactions. This entire *M *and *R *system comprises the (M,R) systems analyzed by Rosen [3] and are said to be organizationally invariant.

**Figure 4:**
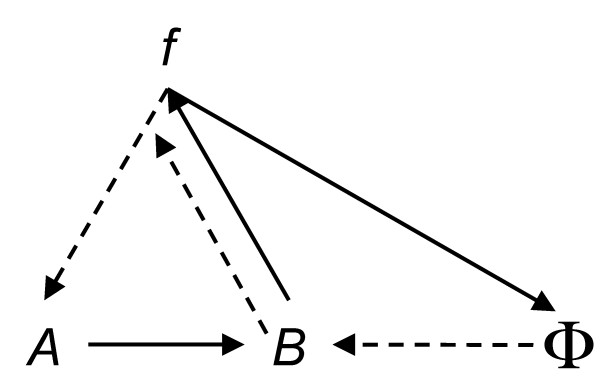
**Commutative mapping relation for M-R systems**.

In order to understand the function of the repair operation, it is important to realize that the domain of the repair is the set {*B*}, so we have Φ: *B *→ *M*(*A*, *B*). The repair comes about at the expense of output from the metabolism and uses metabolism components. An example mapping would formally be written: *b *⟼ Φ (*b*) = *f*, where *f *∈ *M *(keeping the terminology of Rosen and Letelier et al.). We now have

or

our familiar equation derived from anticipatory systems analysis, and can be shown as the commutative mapping in Figure 4[3,21,31]. These are all morphisms of Abelian groups and give us the seemingly infinite regress relation: *f *(*f*) = *f*. This mapping, of course can also be written as *f = f *(*f*) so it is said to be Abelian. But as Cardenas et al. [32] point out, the equation, from a mathematical perspective seems strange, but from a biochemistry perspective it can be rewritten as:

an obviously more acceptable equation. It says that molecules acting on molecules produces molecules.

To avoid the infinite regress we need to recall that the mapping *M *: *A *→ *B *represents *all possible *mappings from inputs to outputs. We impose restrictions, or boundary conditions. First, notice that the set of metabolites {*M*}, and repair-operations {Φ} need to be restricted.

We impose the additional boundary conditions:

Letelier et al. [29] has suggested the further, reasonable, constraint:

|*A*| ≈ |*B*| ≈ |*M *| ≈ |*S|*. This says that the number of reactants | *A *|, is about equal to the number of products | *B *|, and is about equal to the number of enzymes | *M *|, and is about equal to the number of repair operators | *S *|. When we consider the enzymes as the processing machines for the metabolism, then we must also recall that enzymes are produced by the metabolism system. The genome, proteome, metabolome cannot be separated. It is a complex molecular network, and as we will show below the relation |*A*| ≈ |*B*| ≈ |*M*| ≈ |*S| *is not likely valid.

Using the language above, when an enzyme, *M_i _*needs to be repaired, essentially that means there is insufficient quantity of that molecular species for it to participate as a catalyst. The insufficient quantity triggers a threshold to induce some gene to begin a reaction to produce more (a genetic switch in Kauffman's [5] terminology). This is obviously all driven by Le Chatelier's principle: If a chemical systems at equilibrium experiences a change in concentration, temperature, volume or partial pressure, then the equilibrium will shift to counterbalance the change [33]. The complex interactome network is a network of complex irreversible nonequlibrium thermodynamics [34], and summarized by the very-high level commutative mapping shown in Figure 4.

**Figure 5:**
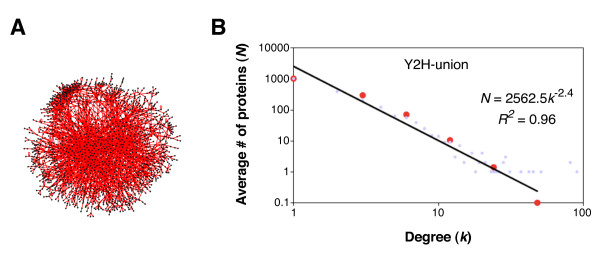
**Yeast protein - protein binary interaction network and the degree distribution plot**. Panel A: protein-protein interaction network for the yeast S. cerevisiae. Panel B: the degree distribution plot showing a power law behavior. Figure reproduced after Yu et al [35].

The above suggests two possible tests of MR-systems theory. First the conditions |*A*| ≈ |*B*| ≈ |*M *| ≈ |*S| *could be investigated by data-mining. The cardinality of these four sets should be about equal. Figure 5 shows the protein-protein interaction network for the yeast, *Saccharomyces cerevisiae *from Y2H experiments and represents "possible" biophysically meaningful interactions. Yu et al. [35] estimate about 18,000 ± 4500 binary protein-protein interactions are possible. Because they did not have all the ORFs for the screening they obtained 2930 binary interactions consisting of 2018 unique proteins giving an average degree, or node valance, of 1.45, computed as a ratio of interactions/proteins.

This of course is only a sketch of the interactome. The full chemical network needs to be closed to efficient causation (i.e., that which is a primary source of change [36]). Further, the full network needs to be at percolation threshold for a self-replicating catalytic network [5,37]. The percolation threshold for a network occurs when the ratio of edges to vertices E/N = 1, for an average degree of 1. This already spells trouble for the cardinality conjecture, |*A*| ≈ |*B*| ≈ |*M*| ≈ |*S| *because the average degree for the incomplete protein-protein interaction network for *S. cerevisiae *is 1.45. This suggests that . If this is correct for the full network, then the mapping relations  are not Abelian.

Though the PPI network graph is not directed, we can still conclude that the mapping is obviously not Abelian because, as shown in the degree distribution, there are some very large hubs. This scale-free observation, which is common for many types of networks, suggests that protein machines are being recruited for more than one metabolic reaction. Biology is a little more complicated than implied by |*A*| ≈ |*B*| ≈ |*M *| ≈ |*S| *and the system dynamics is more complicated than shown in Figure 4.

A second test of the MR-systems theory would be to assemble an autocatalytic set of reactions in a simulation not unlike those by Palsson [14]. Here however, the computational complexity is beyond current systems for anything like a biological cell. But it may be possible to expand the artificial-chemistries/artificial-life simulations similar to Fontana [38,39]. In these simulations we might observe if the relations |*A*| ≈ |*B*| ≈ |*M *| ≈ |*S| *hold, and that the network graph be scale free. The biological MR-system shown in Figure 3 is just a small part of the full interactome [40]. Though for some organisms (e.g. budding yeast) far more details are known than for other organisms, for the most part the full interactome remains a mystery.

If we let percolation threshold in the network,  be the lower bound on the connectivity for molecular networks, we can set the upper bound to the percolation threshold for the adjacency matrix, . Now we have a conjecture that indicates the existing incompletion of the molecular interaction networks. For yeast the number of connections would be 6000^2^/2 ≈ 10^7^.

To expand our parallel analysis of factories and biological cells consider that from a manufacturing perspective, the sets {*A*} and {*B*} are the inputs and outputs to the processing machines. Both biological and manufacturing systems are materially and thermodynamically open. Both are self-regulating, self-repairing dynamical systems. Of course the cell is also a self-replicating system, and as Drexler [41] pointed out, the cell is proof of concept for self-replicating molecular-scale machines. Similarly, self-replicating factories and machines have been described [42].

For cellular systems biology we can view the system as a network of interacting molecular species, with one of the major time lags being diffusion and Brownian motion. Processes can take place reasonably rapidly and Le Chatelier's principle can drive the system dynamics. On the organism level, diffusion and other transport processes can be major time delays, and the dynamics of the organism can be minutes to days to weeks. Similarly, the time lag in manufacturing is far greater between sensing a manufacturing processing component failure (mean time to failure) and actual repair time (mean time to repair). This gives rise to a hysteresis [43]. In the next section we examine more closely some manufacturing networks and compare them with biological networks.

### Manufacturome

Above we described anticipatory systems and M-R systems from mostly a biological perspective, here we will draw further analogies with manufacturing and systems biology. Casti [20,21] explored in detail Rosen's anticipatory systems and MR-systems to manufacturing.

In the mapping , the input to the process *f *is the set {*A*} and the output is the set {*B*}. At the cellular biology scale, the seemingly infinite recursion of the compact version of this map, *f *(*f*) = *f*, can be explained as the fact that the genome, proteome, metabolome are all interrelated. Components and machine parts from the proteome are used in processing the metabolome. Components and machine parts from the proteome are used in resynthesis of the proteome components all the while making use of the metabolome and genome.

In biology the network of interacting proteins, commonly called the interactome, really consists of enzymes and protein inputs/outputs to the metabolism. If we could remove from the protein-protein interactome the inputs and outputs leaving a connected graph or a time sequence list of the enzymes that participate in cell cycle and/or cellular manufacturing, then we would essentially have the following type of linear network:

Where *W *(*t_i_*) represents the metabolites or materials to be processed by *P*_*i*,*j *_during the time period between *i *and *j*. Biochemically *P*_*i*,*j *_would be enzymes. In a manufacturing environment, it would be the processing machine.

Now if we constructed what is called the edge graph for the linear network shown above, would have:

a network of enzymes, or processing machines, as they are used in sequence.

**Figure 6:**
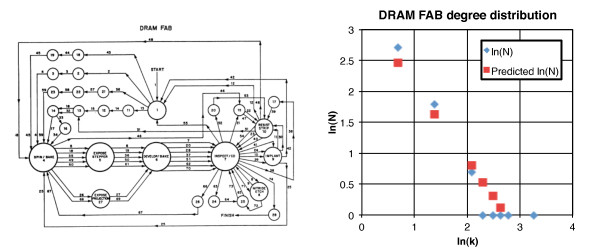
**Network diagram for DRAM integrated manufacturing (left) and the degree distribution plot (right)**.

We have been drawing several parallels between manufacturing and systems biology. Since manufacturing networks are completely known we have an opportunity to explore algebraic graph theory and test algebraic and group theory hypothesis on manufacturing networks that are not possible yet with incomplete biological interactomes. Here we want to point out some network similarities. Figure 6 shows the network graph for DRAM (dynamic random access memory) chip manufacturing [44]. The graph shows a network of the silicon wafer flow from processing step to processing step. This is a network graph showing the sequence of processing steps. It is similar to the above description of the network, *P*_*n*-2,*n*-1 _→ *P*_*n*-1,*n *_and could be laid out linearly; but since the same machine is used for similar processes, hubs are created. Obviously it is a directed graph, information not usually available for interactomes. The figure also shows the degree distribution for this rather small graph, *N = *27.1*k *^-1.2^; *R*^2 ^= 0.94. Notice there are large hubs; the most prominent being the inspection step. The next largest steps are expose stepper and develop/bake. These are lithography steps used to define the regions for transistor location and interconnection. Like the PPI network in Figure 5, processing machines are used over again for different steps in the manufacturing. This is known as a reentrant manufacturing line [43]. But unlike the interactome multiple connections are shown between nodes. For example node 4, 5, 6, and 7 show multiple connections. These multiple connections represent the time dynamics. There are 28 nodes in the network but 148 connections with the nodes balanced in in- and out-degree. The average degree is 5.28 or | *A*|/| *M *| ≈ | *B *|/| *M *| ≈ 5.28. Keeping in mind that the full manufacturome represents other processes, using *M *= 28, the upper bound for the number of edges is 6272. The conjecture |*A*| ≈ |*B*| ≈ |*M *| ≈ |*S| *is valid only when the nodes are replicated the appropriate number of times to capture the dynamics. We never see this in biological interactomes, or manufacturomes because of protein/workstation reuse which embeds the larger cardinality in the edge-count, so the validity of this conjecture remains open, mainly because of incomplete information at this time.

**Figure 7:**
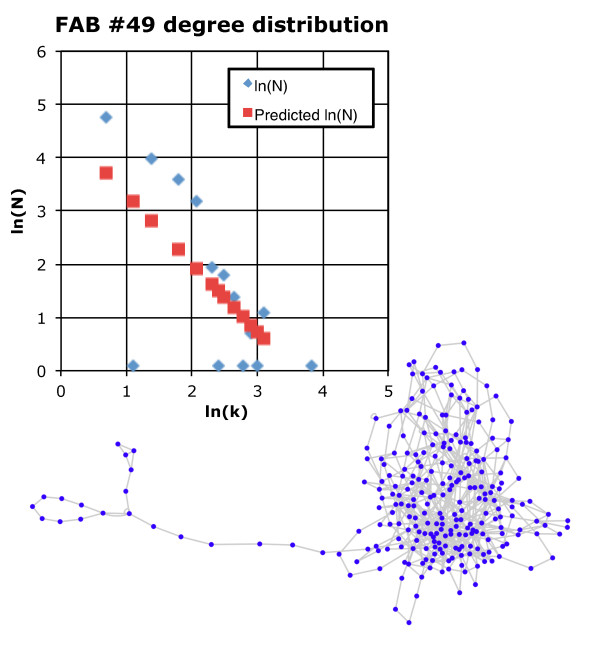
**Network graph for CMOS integrated circuit manufacturing (lower right) and corresponding degree distribution (upper left)**.

Figure 7 shows another manufacturing graph (unpublished data from IBM; 2009), this time for multi-level CMOS integrated circuit manufacturing. The first thing to notice is that this is a far more complicated graph, than the network graph of Figure 6. The circuits being manufactured in the Figure 7 manufacturome are more advanced than the chips produced by the manufacturome of Figure 6 and about 20 times smaller line width. Like the earlier figure, it is a graph showing the processing flow for the silicon wafers from processing step to processing step. As seen in the figure, the degree distribution follows a power-law, because of modularity reuse of processing tools. It contains 259 nodes (processing steps) and 628 edges connecting these nodes. The graph has an average degree of 2.42, and fits the relation *N *= 101.3*k *^-1.3^; *R*^2 ^= 0.44, and given *M *= 259, we get the number of edges, *E = *5.366 × 10^5^.

It is also important to realize that this manufacturome is not an autocatalytic set and the network diagram in Figure 7 is incomplete. We would need to include full factory inputs, outputs, waste stream, and activities of the marketing department, etc. as nodes in the manufacturome. Further, to make MR-system diagrams from the given manufacturome, we would need to show that each machine/process has associated with it, its own repair function in the form of *in situ *signals from the process being analyzed as X-bar (signal average) and R (signal range) charts [43]. These provide clues to the internal dynamics of the manufacturing tool and the process, and allow engineers to make decisions concerning system repair, replacement, or maintenance.

As a final comparison we look at the number of coding genes in the smallest known genome to our CMOS fab. The organism in question, *Mycoplasma genitalium*, contains, 471 coding genes [45]. These breakdown into the following functions: amino acid synthesis, 1; biosynthesis of cofactors, 5; cell envelope 17; cellular processes, 21; central intermediary metabolism, 6; energy metabolism, 31; fatty acid and phospholipids metabolism, 6; purines, pyrimidines, nucleosides and nucleotides synthesis, 19; regulatory functions, 7; replication (DNA degradation, replication, restriction, modification, recombination, and repair), 32; transcription, 12; translation, 101; transport and binding proteins, 34; other categories, 27; unassigned roles, 152. Mushegian and Koonin [46] through comparative bioinformatics deduce that the minimal set is 256 genes. Later work, by Glass et al. [47] from gene knockout experiments, suggests that 382 are the minimal number of coding genes required for life. Whatever the correct number, it is already approximately in the same order of magnitude as the CMOS fab. Which of course does not imply that the fab is a self-replicating system like a cell. With regards to the "minimal" cell of 256 genes (*M *= 256), the upper bound for the number of edges in the full molecular network would have about *E = *5.24 × 10^5^.

## Conclusions

In summary, the development of main equations for anticipatory systems and metabolism-repair systems are similar for manufacturing systems and cellular biology. The fact that these two disparate domains are so tightly coupled by similar mathematics suggests these concepts are indeed at the boundary of living and nonliving. Of course the coupling could also be a coincidence because of the "unreasonable effectiveness of mathematics in the natural sciences" [48].

We reviewed the basis for the self-referential relation *f = f *(*f*) and found that the boundary condition |*A*| ≈ |*B*| ≈ |*M *| ≈ |*S| *can't be valid, but is likely only a lower bound. We suggest that the upper bound is the percolation threshold for the adjacency matrix of the molecular network, and compute these lower and upper bounds for *S. cerevisiae, M. genitalium *and two integrated circuit manufacturing lines. Further the relation *f = f *(*f*) is not likely Abelian so theoretical understanding of metabolic closure of living cells remains and open question.

## Competing interests

The authors declare that they have no competing interests.

## Authors' contributions

EAR did the research, did most of the calculations and wrote the first draft. JZC provided data for the manufacturome and assisted in analysis of the IBM data. JAT assisted in presenting and integrating the material into the manuscript and coordinating the project. All authors read and approved the final manuscript.

## List of abbreviations

**ADP**: Adenosine diphosphate; **ATP**: Adenosine triphosphate; **CMOS**: Complementary metal-oxide-semiconductor; **DNA**: Deoxyribonucleic acid; **DRAM**: Dynamic random access memory; **Fru6P**: Fructose 6-phosphate; **G6P**: Glucose-6-phosphate; **HXK**: Hexokinase; **M-R**: Metabolism-repair; **MAP**: Mitogen-activated protein; **PGI**: Phosphoglucose isomerase; **PPI**: Protein-protein interaction.
